# Prevalence of obesity and overweight and associated factors among financial institution workers in Accra Metropolis, Ghana: a cross sectional study

**DOI:** 10.1186/s13104-015-1590-1

**Published:** 2015-10-23

**Authors:** Prince N. O. Addo, Kofi M. Nyarko, Samuel O. Sackey, Patricia Akweongo, Bismark Sarfo

**Affiliations:** Department of Epidemiology and Disease Control, School of Public Health, College of Health Sciences, University of Ghana, P. O. Box LG 13, Legon, Accra, Ghana; Non Communicable Diseases Control Program, Ghana Health Service, Box KB 493, Korle-Bu, Accra, Ghana

**Keywords:** Obesity, Overweight, Body Mass Index, Financial Institution, Physical Activity, Cross-sectional, Work place

## Abstract

**Background:**

Certain professions are associated with low physical activity. Workers in such professions spend the most part of their adult working lives less engaged in physical activity if they don’t consciously exercise outside of working hours. This increases their risk of obesity and its associated diseases. This study determined the prevalence of obesity and overweight and associated factors among workers of a financial institution in Accra Metropolis, Ghana.

**Methods:**

A cross-sectional study was conducted among 180 workers of a financial institution in Accra using the World Health Organization’s STEPS (STEPwise approach) instrument for non-communicable disease risk factor surveillance. Relevant sociodemographic information were recorded and BMI was computed for each respondent.

**Results:**

The overall prevalence of obesity and overweight among the bank workers was 55.6 % (17.8 % obese and 37.8 % overweight). After adjusting for other variables, physical activity (OR = 0.34, 95 % CI = 0.13–0.89, p = 0.03), alcohol consumption (OR = 3.00, 95 % CI = 1.35, 6.68, p = 0.007), marital status (OR = 2.74, 95 % CI = 0.96–7.85, p = 0.04), sex (OR = 2.78, 95 % CI = 1.23–6.33, p = 0.01), and age (OR = 1.10, 95 % CI = 1.01–1.20, p = 0.036) were significantly associated with obesity and overweight.

**Conclusion:**

Being physically inactive, consumption of alcohol, being married and a female, in addition to old age, increase the risk of obesity and overweight significantly. These factors should inform policy makers in developing strategies to reduce the burden of obesity and overweight among this category of workers.

## Background

The human body needs energy to function and food is the source of this energy. The body stores excess energy mainly in the form of fats [1, 2]. According to the World Health Organization (WHO), when an individual’s health is impaired by excessive body fat, that person is said to be obese.

Obesity, which is the Body Mass Index (BMI) greater than or equal to 30 kg/m^2^ of an individual is a major risk factor for several non-communicable diseases [3, 4,5]. Notable among these are diabetes (type 2), sleep disorders, depression, anxiety, cancers and many cardiovascular diseases [6]. As BMI increases, the risks of these diseases also increase [3]. In 2013, obesity was classified as a disease by the American Medical Association to get physicians to pay more attention to this condition [7].

World Health Organization updated report [8], shows that the prevalence of obesity worldwide has increased tremendously since 1980. The report indicates that 39 and 13 % of people 18 years and above were overweight and obese respectively in 2014. Obesity and overweight kills more people than underweight in many countries, and these countries account for 65 % of the global population [8].

Obesity is rapidly establishing itself as a public health problem in several developing countries [9, 10, 11] with urban communities having higher prevalence of obesity, particularly among those of higher socioeconomic status [6, 12, 13, 14].

A substantial decrease in physical activity levels and energy expenditure, coupled with an increase in energy intake has been reported as the main factors promoting obesity [15]. The World Health Organization reported that the principal reason for this excess weight problem is an energy imbalance between calories consumed and calories expended. Increasing intake of foods high in energy and decreasing level of physical activity due to increasing urbanization, changing modes of transportation and sedentary working environments account for this energy balance [8].

Certain individual level factors and lifestyles have been known to be major risk factors for obesity. Obesity (BMI ≥30 kg/m^2^) and overweight (BMI <30 kg/m^2^ but ≥25 kg/m^2^) have been reported to be higher among poor urban women and women with basic or no education. A study conducted in Ghana in 2005 revealed that obesity is more common among those who are married, and also among those who are employed [16]. Alcoholic consumption and physical inactivity have all been associated with obesity and overweight [16].

Certain occupations predispose individuals to sedentary lifestyles and some of these are white collar jobs characterized by sitting for long periods of time. For instance, workers of financial institutions (banks) who serve in the banking hall (tellers and customer service personnel) and back office, spend the greater part of the day sitting down due to the nature of their work [17]. Eventually, these individuals will spend the most part of their adult working lives less engaged in physical activity if they don’t consciously involve themselves in physical or sporting activities outside of working hours [18].

Therefore, workers in such institutions become susceptible to developing obesity and or overweight which could predispose them to chronic diseases associated with physical inactivity. A study carried out in India reported a much higher prevalence of hypertension (which was positively correlated to obesity/overweight) among bank employees than among the general urban population in the country [19]. This could be due to changes in the metabolism of these employees as they continually sit for long hours [20, 21].

In recent years, there has been an increase in the number of banking institutions (both public and private) in Ghana; hence the number of people working in these institutions is also expected to increase. This could contribute to the increasing prevalence of obesity and overweight in the country, with its related health implications. Studies involving employees of financial institutions and the health risks associated with their job are rare in Ghana and other developing countries. Health of employees must be protected, so they would continue to be productive to contribute to the growth the country. This study determined factors associated with obesity and overweight prevalence among workers of a financial institution in Accra Metropolis of Ghana.

## Methods

### Study design

The study was a cross-sectional study using a structured questionnaire and physical measurements of height and weight among financial institution employees in Accra metropolis.

The WHO’s STEP-wise approach to surveillance of non-communicable disease risk factors was employed in the study. STEPS is a standardized tool applicable in any setting for monitoring modifiable risk factors for non-communicable disease [22, 23]. Level of physical activity was assessed with the help of the General Practice Physical Activity Questionnaire (GPPAQ).

### Study site

Accra is the capital city of the Greater Accra Region of Ghana and is also the capital city of Ghana. It is the largest city of Ghana, with an estimated population of 2.27 million. Accra Metropolis is one of the ten (10) districts in the Greater Accra Region and is highly urbanized. It is the 11th largest metro area in Africa, with about 4 million inhabitants [24].

Being the capital city of Ghana, there are many business activities going on and it is the preference for many entrepreneurs and financial institutions who either want to establish or expand their businesses.

Many studies conducted in Africa have reported higher prevalence of obesity in urban areas [6, 25, 26], hence the reason for choosing Accra Metropolis for this study.

This study was conducted in the Accra branches of one of the fastest growing financial institutions in Ghana. The study population was made up of mainly employees whose job description requires them to sit for the most part of the working day. To be included in the study, an employee from the selected branch of the financial institution must be a teller (cashier), customer service personnel or a “back office” staff (managers, supervisors and other supporting staff). These employees sit for the most part of the working day. All other workers of the financial institution who fell outside these groups were excluded from the study.

### Sample size and sampling

Sample size of 172 was calculated with Stata 11 software using proportion of obesity and overweight as 30 %, at 95 % confidence interval and a 5 % margin of error. Allowing for a 5 % non-response rate, the effective sample size was adjusted to a total of 180 participants.

The study was carried out in all eight branches of the financial institution in Accra. The list of employees in those branches was obtained from the human resource department of the bank. The sample size for each branch was computed by dividing the total population of that branch by the overall population of workers in all eight branches of the bank and multiplying the results by 180 (total sample size). At each branch, a list of employees was obtained and arranged in alphabetical order by surname. This total list was divided by the sample required for that branch to get the selection interval. At each branch, selection of participants was done using the attendance register for the day. This register was used to systematically select participants (first name on the register, third name, fifth name, in that order). A total of 180 employees (92 males and 88 females) participated in the study.

### Ethical approval

Ethical approval was obtained from the Ghana Health Service and the Noguchi Memorial Institute for Medical Research (University of Ghana) Ethics Review Committee. Written permission was sought from the management of the bank before commencement of the study. Participants were asked for their verbal consent before they were interviewed. They were informed of the voluntary nature of participation and were assured of confidentiality. Participants found to be obese were educated on the health implications of obesity and how to reduce their weight through regular physical activity and healthy eating habits. Obese participants were also introduced to dieticians and experts in physical activity to help them maintain a healthy weight.

## Data collection

### Sociodemographic variables

Ages, sex, educational level, marital status, years with institution and sedentary work were assessed with the help of a structured questionnaire.

Height measurements were done using a stadiometer (Detecto model). Each subject was made to remove his/her footwear, stand feet together and arms at the sides. Subject stood with heels, buttocks and upper back against the straight edge in a complete upright position. Measurement of height was taken in centimeters and then expressed in meters.

Weight was measured with the help of an electronic bathroom scale (Omron model) with a maximum capacity of 150 kg. Each participant was made to remove his/her footwear, stand feet together and arms at the sides on the scale. Data were collected between 7am and 9am in the morning.

### Physical activity

Level of physical activity was assessed with the help of the General Practice Physical Activity Questionnaire (GPPAQ). GPPAQ was developed by the London School of Hygiene and Tropical Medicine as a validated short measure of physical activity for adults (16–74 years). The input of a participant’s physical activity information into the online version of the questionnaire generates the activity level of the individual [27]. It provides a 4-level Physical Activity Index (PAI), categorizing subjects as:

#### Active

Sedentary job and ≥3 h physical exercise and/or cycling per week OR standing job and 1–2.9 h physical exercise and/or cycling per week OR physical job and some but <1 h physical exercise and/or cycling per week OR heavy manual job.

#### Moderately active

Sedentary job and 1–2.9 h physical exercise and/or cycling per week OR standing job and some but <1 h physical exercise and/or cycling per week OR physical job and no physical exercise or cycling.

#### Moderately inactive

Sedentary job and some but <1 h physical exercise and/or cycling per week OR standing job and no physical exercise or cycling.

#### Inactive

Sedentary job and no physical exercise or cycling [28].

A physical activity index less than Active suggests the need for a brief intervention supporting behavioral change to increase physical activity.

### Sedentary work

Sedentary work was assessed with the help of the General Practice Physical Activity Questionnaire (GPPAQ).

### Alcohol intake

Alcohol intake was assessed with the help of questionnaire. This study was interested in identifying the differences in obesity/overweight prevalence comparing respondents who take alcoholic beverages at least once a week to respondents who do not take alcohol at all. Quantity of alcohol taken was not considered.

Body Mass Index (BMI) was classified according to WHO international classification of adult BMI. The classification is as follows: Underweight (<18.50 kg/m^2^), Normal range (18.50–24.99 kg/m^2^), Overweight/Pre-obese (25.00–29.99 kg/m^2^) and Obese (≥30 kg/m^2^), [29].

### Statistical analysis

Analysis was carried out using Stata 11 for windows. Data was checked for missing values, consistency, possible data entry errors and normality. Frequencies were performed for all variables to know their distribution and range. Age was categorized as “<25, 25–34, 35–44, 45–54”. Years with institution was categorized as <1, 1–3 and >3 years. Sex was in two categories (male or female), educational level was in four categories (none, junior high, senior high and tertiary, marital status was in four categories (single, married, cohabiting and divorced), sedentary work was either “yes” or “no” and alcohol consumption was put into two categories (yes or no).

Body Mass Index (BMI) was the main outcome of interest (dependent variable) in the study. Independent variables included sex, age in years, marital status, alcohol consumption, educational level, and years with institution. Measures of central tendency such as means and standard deviations were computed for continuous variables.

Prevalence of obesity and overweight (BMI) were estimated as percentages with their corresponding 95 % confidence intervals. Association between BMI and each of the other independent variables was assessed using Chi square test at a 5 % level of significance. Bivariate analyses of BMI with each independent variable were carried out using logistic regression. For this analysis, the 4 categories of physical activity were collapsed into 2 categories: inactive (inactive + moderately inactive) and active (active + moderately active). The underweight and normal groups of BMI were collapsed = under/normal and the overweight and obese groups were collapsed = overweight/obese. Age was assessed by comparing participants in the highest age group (45–54) to those in the lowest age group (<25). Marital status was assessed by comparing married respondents to single respondents.

All statistically significant variables from the bivariate logistic regression were added to the multiple logistic regression model.

## Results

One hundred and eighty (180) employees of a financial institution in Accra Metropolis participated in the study.

### Characteristics of participants

This study recruited 51.1 % males and 48.9 % females. The mean age was 32.2 years (±6.9) and it ranged from 19 to 54 years (Table 1). The mean height of respondents was 1.68 (±0.13) m, mean weight was 80.10 (±15.20) kg and mean BMI was 26.90 (±3.70) kg/m^2^. Most of them (58.3 %) were aged 25–34 years. Majority of the participants (81.7 %) had attended a tertiary institution. Fifty-five percent (55 %) of them were married and 40 % were single. Most of the workers (57.2 %) had been with the institution for more than 3 years. The job description of 85 % of participants required them to sit (sedentary) for the most part of the working day. Fifty-seven percent (57 %) of the workers consume alcohol at least once per week. The percentage of participants who were overweight was 37.8 %, whilst 17.8 % of them were obese. Only 16.7 % of participants were physically active (Table 1). The percentage of respondents who were physically inactive was higher in the overweight/obesity group (66.7 %) than in the underweight/normal group (33.3 %), (Table 1).

**Table 1:** Characteristics of respondents (n = 180)

Variable	Participants	Under/normal	Overwt/obese	Chi square
	n (%)	n (%)	n (%)	(*p* value)
Sex				*χ* ^2^ = 0.11
Male	92 (51.1)	42 (45.6)	50 (54.4)	p = 0.74
Female	88 (48.9)	38 (43.2)	50 (56.8)	
Age				*χ* ^2^ = 23.74
<25	13 (7.2)	11 (84.6)	2 (15.4)	p < 0.0001
25–34	105 (58.3)	55 (52.4)	50 (47.6)	
35–44	53 (29.4)	13 (24.5)	40 (75.5)	
45–54	9 (5.0)	1 (11.1)	8 (88.9)	
Educational level				*χ* ^2^ = 3.05
None	4 (2.2)	2 (50.0)	2 (50.0)	p = 0.38
Junior high	7 (3.9)	3 (42.9)	4 (57.1)	
Senior high	22 (12.2)	6 (27.3)	16 (72.7)	
Tertiary	147 (81.7)	69 (46.9)	78 (53.1)	
Marital status				*χ* ^2^ = 25.77
Single	72 (40.0)	48 (66.7)	24 (33.3)	p < 0.001
Married	99 (55.0)	28 (28.3)	71 (71.7)	
Cohabiting	3 (1.7)	2 (66.7)	1 (33.3)	
Divorced	6 (3.3)	2 (33.3)	4 (66.7)	
Years with institution				*χ* ^2^ = 11.27
<1 year	36 (20.0)	24 (66.7)	12 (33.3)	p = 0.004
1–3 years	41 (22.8)	20 (48.8)	21 (51.2)	
3 years or more	103 (57.2)	36 (35.0)	67 (65.0)	
Sedentary work				*χ* ^2^ = 0.71
No	27 (15.0)	10 (37.0)	17 (63.0)	p = 0.40
Yes	153 (85.0)	70 (45.8)	83 (54.2)	
Alcohol				*χ* ^2^ = 4.81
No	76 (42.2)	41 (54.0)	35 (46.0)	p = 0.03
Yes	104 (57.8)	39 (37.5)	65 (62.5)	
Physical activity				*χ* ^2^ = 9.61
Inactive	84 (46.6)	28 (33.3)	56 (66.7)	p = 0.03
Moderately inactive	36 (20.0)	17 (47.2)	19 (52.8)	
Moderately active	30 (16.7)	16 (53.3)	14 (46.7)	
Active	30 (16.7)	19 (63.3)	11 (36.7)	

Alcohol consumption = at least once/week

### Factors associated with obesity and overweight

Variables that showed statistically significant association with obesity and overweight using Chi square test (p < 0.05) were age, marital status, years working with the bank, alcohol consumption and level of physical activity (Table 1).

Logistic regression was subsequently performed to see the strength of association between each of these risk factors and obesity or overweight (Table 2). Age was still significantly associated with being obese or overweight (Crude OR = 3.54, 95 % CI 2.03–6.16, p < 0.001). Married participants showed an increased risk of obesity or overweight in comparison to singles (OR = 5.07, 95 % CI 2.63–9.78, p < 0.001). Respondents who had worked for 3 years and above were 3.72 times more likely to be overweight or obese than those who had worked for <1 year (Crude OR = 3.72, 95 % CI 1.67–8.31, p = 0.001). Participants who take alcohol at least once per week were at a higher risk of becoming obese or overweight (Crude OR = 1.95, 95 % CI 1.07–3.56, p = 0.03). Physically active respondents had a 60 % reduced risk of obesity/overweight compared to those who were not physically active (Crude OR = 0.40, 95 % CI 0.18–0.89, p = 0.03).

**Table 2:** Factors significantly associated with obesity and overweight

Variable	Overwt/obese	P value	Overwt/obese	P value
	Crude OR (95 % CI)		Adjusted OR (95 % CI)	
Sex		0.734		0.014
Male	1		1	
Female	1.11 (0.61–1.99)		2.78 (1.23–6.33)	
Age		<0.001		0.036
<25	1		1	
45–55	3.54 (2.03–6.16)		1.10 (1.01–1.20)	
Marital status		<0.001		0.04
Single	1		1	
Married	5.07 (2.63–9.78)		2.75 (0.96–7.85)	
Years with institution		0.001		0.27
<1 year	1		1	
3 years or more	3.72 (1.67–8.31)		1.74 (0.64–4.72)	
Alcohol consumption		0.03		0.007
No	1		1	
Yes	1.95 (1.07–3.56)		3.00 (1.35–6.68)	
Physical activity		0.025		0.03
Inactive	1		1	
Active	0.4 (0.18–0.89)		0.34 (0.13–0.89)	

The following variables remained significantly associated with obesity and overweight in the multivariate logistic regression analysis: sex, age, marital status, alcohol consumption and physical activity (Table 2).

## Discussion

The overall prevalence of obesity and overweight among the bank workers was 55.6 % (17.8 % obese and 37.8 % overweight) as against the total prevalence of overweight and obesity of 30 % indicated in the Ghana Demographic and Health Survey report [30]. A study also conducted in Accra by Aryeetey and Ansong reported an overall prevalence of obesity and overweight of 56 % [31]. Many other studies carried out in Africa have reported higher prevalence of obesity and overweight in urban areas [32, 33, 25, 26]. The high prevalence of obesity and overweight among the bank employees could be attributed to less physical activity due to the sedentary nature of their work resulting in weight gain [34].

The percentage of males who were overweight/obese was almost the same as that of females (Table 1). After adjusting for other variables, the risk of obesity and overweight among females was twice that of males. This finding is in line with Case and Menendez [35] reports which sought to find out the underlying causes of the disparities in obesity comparing women to men using data from a South African township. The report indicated that poverty in childhood and greater access to resources in adulthood, lead women to be at significantly greater risk of obesity than men and that, women aspire to have larger body sizes and therefore use their resources to acquire it [35]. Another cross-sectional study conducted in Khayelitsha in Cape Town-South Africa reported a higher prevalence of overweight/obesity among women than men and concluded that men had about 90 % reduced risk of obesity/overweight [36]. Findings from a review of data on Body Mass Index explained that overweight/obesity among women in developing countries is largely seen as a sign of beauty and affluence, and could be related to the higher proportion of obese women than men in such regions [37]. This study found a strong association between obesity and age, with the prevalence of obesity increasing as aged increased. This is in line with findings from a review of data on the prevalence of adult obesity/overweight from the World Health Organization’s Global Database on Body Mass Index which showed an increasing trend of prevalence of adult obesity with age. The peak of this trend is estimated to be 40–50 years in many developing countries in contrast to 50–60 years in most developed countries [37]. A review done in the U.S also showed that the combined prevalence of obesity and overweight among men and women 20 years and above increased with age [38]. A recent national data on prevalence of obesity among children, adolescents and adults revealed age differences in obesity prevalence by sex. Prevalence of obesity increased by age among women but no such difference in prevalence by age was found among men [39]. Age is one of the strongest risk factors that predispose an individual to the development of overweight/obese in most populations [40].

A cross-sectional study that employed data from national health interview surveys from nineteen European countries reported higher prevalence of obesity and overweight among people with lower educational status [41]. This current study did not find any association between educational attainment and risk of obesity or overweight. This could result from the fact that majority of respondents were in the same educational bracket (tertiary). The relationship between age, gender, educational attainment (socio-economic status) and obesity is a complex and dynamic one. Authors in another study that found an inverse relationship between higher education and obesity argued that adjustment for age might lead to smaller differences in obesity prevalence between educational classes [40].

Findings from this study show that, the odds of obesity/overweight increased about 2.75 times comparing people who are married to those who are single. This supports findings from a Spanish cross-sectional study with a much larger sample-size. The study reported 1.69 odds of obesity comparing married people to single people [42]. A study that investigated whether romantic partnership and duration of cohabitation are related to obesity and obesity-promoting behaviors concluded that single individuals who start dating, then cohabiting and later married were more likely to become obese. Obesity-related behaviors were also strongest among married couples and couples who had lived together for 2 years or more [43]. Data from the first national obesity prevalence survey among Greek adults was used in a study aimed at determining the association of obesity and overweight with marital status and educational level. A higher risk of obesity and overweight was found in married men and women than their respective single counterparts [44]. Sobal et al. however argue that, associations of marriage with body weight appear to be at least partly contingent upon gender and ethnicity, which may reflect larger societal patterns of involvement in marriage, commitment to family, and body-weight norms and expectations [45].

The current study found a higher prevalence of obesity and overweight among sedentary workers. Sedentary work has been shown to increase the risk of obesity and overweight [34]. Sedentary behavior has also been shown to adversely affect health outcomes [46, 47, 48]. A strong association between sedentary work and physical inactivity was also found in this study. The findings from this study also indicated that the risk of obesity/overweight increases with the number of years spent working with the banking institution, with people who had worked for 3 years or more having an increased risk of obesity/overweight in comparison to those who had worked for less than 1 year. However, this relationship was not significant in the adjusted model.

Findings from this study show that, alcohol consumption increases the risk of obesity/overweight three times comparing those who take alcohol to those who do not take alcohol. Ghana is one of the countries that participated in the 2003 World Health Survey on health status and health system responsiveness. Findings from this survey showed a higher proportion of alcohol consumers being overweight/obese [16]. Another study conducted among over 35,000 Dutch adolescents confirms this finding [49]. However, a prospective cohort study conducted among American women showed a reduction in the risk of obesity and/overweight among women who consumed little to moderate amount of alcohol as compared to non-drinkers [50]. The age, gender, weight, height and activity level of an individual determines the number of calories needed to maintain a healthy weight. Consuming more calories than the body needs leads to weight gain. Alcohol can increase the risk of weight gain by the calories it provides and by increasing ones appetite for food [51]. A large number of studies have also reported that alcohol consumption does not necessarily lead to weight gain [52, 53]. The underlying reason for this is unclear. Some researchers say that energy from alcohol is not efficiently used. Others also indicate that alcohol appears to bring about an increased metabolic rate, resulting in more calories being burnt than stored as fat [54, 55].

Physical inactivity was one of the most important risk factors for obesity and overweight identified in this study. Physically active participants had a 66 % reduced risk of obesity and overweight in comparison to participants who were not physically active. The percentage of non-active (inactive, moderately inactive and moderately active) respondents was quite high (83.3 %), leaving much smaller percentage of physically active respondents (16.7 %). The percentage of overweight/obese respondents increased as the level of physical activity decreased (Table 1). Many other studies have also shown an inverse relationship between obesity and physical activity [34, 8, 54, 55]. Occupational sitting time is therefore a major contributor to the obesity epidemic [56, 57, 58]. This is therefore an area that needs attention in mitigating health related conditions resulting from long hours of sitting in the office.

### Limitation of the study

The third step (biochemical measurements) of the WHO STEPWISE Approach for Non-Communicable Disease surveillance was not carried out in this study due to inadequate technical staff and insufficient funds to carry out the work involved. However, the STEPS 1 and 2 provide sufficient evidence to validate any findings and conclusions of the study. Also, there is the limitation with self reporting.

## Conclusion

Being physically inactive, consumption of alcohol, being married and a female, in addition to old age, increase the risk of overweight and obesity significantly. These factors should inform policy makers in developing strategies to reduce the burden of overweight and obesity among this category of workers.

## Recommendations

Interventions such as sports, keep fit clubs, gym membership, aimed at promoting physical activity must be implemented among sedentary workers. These activities could be spearheaded by the management of institutions such as banks with large proportions of sedentary workers. Local and international health organizations must intensify their advocacy for physical activity, especially in the work place and among the general populace.
